# Draft Genome Sequence of *Plantibacter* *flavus* Strain 251 Isolated from a Plant Growing in a Chronically Hydrocarbon-Contaminated Site

**DOI:** 10.1128/genomeA.00276-17

**Published:** 2017-04-27

**Authors:** Rhea Lumactud, Roberta Fulthorpe, Vladimir Sentchilo, Jan Roelof van der Meer

**Affiliations:** a Department of Physical and Environmental Sciences, University of Toronto Scarborough, Toronto, Ontario, Canada; b Department of Fundamental Microbiology, University of Lausanne, Lausanne, Switzerland

## Abstract

*Plantibacter flavus* isolate 251 is a bacterial endophyte isolated from an *Achillea millefolium* plant growing in a natural oil seep soil located in Oil Springs, Ontario, Canada. We present here a draft genome sequence of an infrequently reported genus *Plantibacter*, highlighting an endophytic lifestyle and biotechnological potential.

## GENOME ANNOUNCEMENT

*Plantibacter flavus* isolate 251 was isolated from an *Achillea millefolium* plant growing in a chronically hydrocarbon-contaminated site located in Oil Springs, Ontario, Canada. This isolate was found to be common among all sampled plants. Using a colorimetric assay and through their ability to reduce tetrazolium dye, this isolate was found to potentially mineralize petroleum hydrocarbon substrates, being strongly positive for octanol, toluene, and naphthalene, and weak for kerosene and motor oil, on the basis of purple color appearance. Using gas chromatography mineralization, *P. flavus* 251 was able to degrade 37% each of toluene and naphthalene substrates (1). The species *Plantibacter flavus* was first reported by Behrendt et al., was found to be associated with grass, and was isolated from the phyllosphere and litter layer (2).

DNA was subjected to sequencing on Pacific Biosciences RSII single-molecule real-time (SMRT) cells sequencing platform at the Lausanne Genomic Technologies Facility (University of Lausanne, Switzerland). A total of 51,440 reads were obtained with an average length of 6,633 bp and average quality of 0.86. The PacBio reads were assembled *de novo* using SMRT Analysis suite and the HGAP.3 algorithm. The assembled contiguous chromosomal sequence was 4,211,582 bp, with a G+C content of 69.2%. Annotation was conducted using RAST Web server (3, 4), generating 3,902 coding sequences, of which 41% were classified in 394 subsystems.

The *P. flavus* 251 contained 555 features associated with the subsystem carbohydrates, of which 136 were for monosaccharides, 114 were for disaccharides and oligosaccharides, and nine were for polysaccharides, 50 were for amino sugars, and 37 were for one-carbon metabolism; this highlights an endophytic lifestyle targeted to plant sugars. The genome contained no distinguishable features for any of the regular secretion systems (e.g., types I to VIII), but showed 84 features for ABC transporters. Two prophages were found, as were genes for arsenic, mercury, and cadium-zinc-cobalt resistance. No genes were found for bacterial motility or chemotaxis. Pathways for tricarboxylic acid metabolism, glycolysis and pyruvate metabolism were complete. Despite demonstrated utilization of toluene, no known genes for classical toluene, naphthalene, benzoate or biphenyl metabolism were found in the *P. flavus* 251 genome, except three genes for putative salicylate hydroxylase and a gene for a putative catechol-2,3-dioxygenase. The genes for 1-aminocyclopropane-1-carboxylate (ACC) deaminase, which helps plant tolerate contaminant-induced stress by decreasing stress ethylene production (5), were detected in the *P. flavus* 251 genome but were not incorporated in the subsystems analysis.

This newly sequenced *P. flavus* 251 genome is one of the very few reported *Plantibacter* genomes. There is still very limited genomic information on this genus. Given that no known hydrocarbon-degrading genes were found despite showing phenotypic abilities on metabolism of hydrocarbon substrates, this genome likely possesses novel biodegradation enzymes. It is hoped that this genome will provide insights in exploring hydrocarbon degradation and plant growth promotion potentials of a plant-associated microbe.

### Accession number(s).

This whole-genome sequencing project has been deposited at GenBank under accession no. CP019402. The version described in this paper is the first version.
